# Increased Expression of SRSF1 Predicts Poor Prognosis in Multiple Myeloma

**DOI:** 10.1155/2023/9998927

**Published:** 2023-05-10

**Authors:** Jiawei Zhang, Zanzan Wang, Kailai Wang, Dijia Xin, Luyao Wang, Yili Fan, Yang Xu

**Affiliations:** ^1^Department of Hematology, The Second Affiliated Hospital, Zhejiang University School of Medicine, Hangzhou 310009, China; ^2^Zhejiang University Cancer Institute, Key Laboratory of Cancer Prevention and Intervention, China National Ministry of Education, The Second Affiliated Hospital, Zhejiang University School of Medicine, Hangzhou 310009, China; ^3^Department of Hematology, Ningbo First Hospital, Ningbo 315010, China; ^4^Zhejiang Provincial Key Laboratory for Cancer Molecular Cell Biology, Life Sciences Institute, Zhejiang University, Hangzhou, Zhejiang 310058, China

## Abstract

**Background:**

Multiple myeloma (MM) is a clonal plasma cell disorder which still lacks sufficient prognostic factors. The serine/arginine-rich splicing factor (SRSF) family serves as an important splicing regulator in organ development. Among all members, SRSF1 plays an important role in cell proliferation and renewal. However, the role of SRSF1 in MM is still unknown.

**Methods:**

SRSF1 was selected from the primary bioinformatics analysis of SRSF family members, and then we integrated 11 independent datasets and analyzed the relationship between SRSF1 expression and MM clinical characteristics. Gene set enrichment analysis (GSEA) was conducted to explore the potential mechanism of SRSF1 in MM progression. ImmuCellAI was used to estimate the abundance of immune infiltrating cells between the SRSF1^high^ and SRSF1^low^ groups. The ESTIMATE algorithm was used to evaluate the tumor microenvironment in MM. The expression of immune-related genes was compared between the groups. Additionally, SRSF1 expression was validated in clinical samples. SRSF1 knockdown was conducted to explore the role of SRSF1 in MM development.

**Results:**

SRSF1 expression showed an increasing trend with the progression of myeloma. Besides, SRSF1 expression increased as the age, ISS stage, 1q21 amplification level, and relapse times increased. MM patients with higher SRSF1 expression had worse clinical features and poorer outcomes. Univariate and multivariate analysis indicated that upregulated SRSF1 expression was an independent poor prognostic factor for MM. Enrichment pathway analysis confirmed that SRSF1 takes part in the myeloma progression via tumor-associated and immune-related pathways. Several checkpoints and immune-activating genes were significantly downregulated in the SRSF1^high^ groups. Furthermore, we detected that SRSF1 expression was significantly higher in MM patients than that in control donors. SRSF1 knockdown resulted in proliferation arrest in MM cell lines.

**Conclusion:**

The expression value of SRSF1 is positively associated with myeloma progression, and high SRSF1 expression might be a poor prognostic biomarker in MM patients.

## 1. Introduction

Multiple myeloma (MM) is characterized by abnormal proliferation of clonal plasma cells that produce monoclonal immunoglobulin or M protein in bone marrow, leading to organ dysfunctions, such as hypercalcemia, renal failure, anemia, and bone lesions [1, 2]. Monoclonal gammopathy of undetermined significance (MGUS) is a premalignant stage, and approximately, 0.5–1% of MGUS can transform into MM per year [3]. Between MGUS and MM, smoldering multiple myeloma (SMM) represents an intermediate, asymptomatic condition without the SLiM features, which stand for sixty, light chain ratio, MRI, but at a higher risk of progression to MM [4]. Plasma cell leukemia (PCL) is an aggressive MM variant defined by the presence of 5% or more circulating plasma cells in peripheral blood smears in patients with symptomatic MM [5–7].

Alternative splicing generates different RNA isoforms and increases protein diversity to ensure normal development. Thus, abnormal regulation of mRNA splicing may produce altered proteins with oncogenic potential that contribute to cancer development [8, 9]. Previous studies have demonstrated that alternative splicing mediated by mutant splicing regulators could drive the initiation and progression of hematological malignancies [10–13]. The serine/arginine-rich splicing factor (SRSF) family has 12 members that share the conserved serine/arginine (SR) domain and have been reported to be essential for development [14]. Loss of SRSF genes leads to embryonic lethality and organ failure [15–17]. Previous studies have shown that SRSF1 is a potent oncogene and upregulated in many solid tumors, including breast, lung, and liver cancer [18–20]. Additionally, studies about the role of SRSF1 in normal hemopoiesis and hematological malignancies have been emerging as well. For example, SRSF1 is a critical post-transcriptional regulator in the late stage of thymocyte development [21, 22]. Upregulated SRSF1, with the cooperation of PRTM1, acts as an adverse factor in pediatric acute lymphoblastic leukemia [23]. In chronic myeloid leukemia, overexpression of SRSF1 resulted in impaired imatinib sensitivity via BCR-ABL1 and cytokine-mediated signaling pathways [24]. However, the role of SRSF1 in MM is still unclear.

In order to explore the role of SRSF1 in MM, we investigated the relationship between SRSF1 expression and MM progression, ISS stages, amplification of 1q21, relapse status, and prognosis. We also explore the possible underlying mechanisms of SRSF1 in MM. To make our results more credible, we detected SRSF1 expression in clinical samples and reduced SRSF1 expression in MM cell lines to investigate the role of SRSF1 in MM development. Using a combination of comprehensive bioinformatic and experimental analysis, we conclude that SRSF1 is an unfavorable prognostic indicator in MM and essential for MM development.

## 2. Methods

### 2.1. Data Sources

In this study, we selected 11 datasets from the Gene Expression Omnibus (GEO) (http://www.ncbi.nlm.nih.gov/geo) to explore the role of SRSF1 in MM. A total of 3928 samples were included, among them were 32 normal controls, 62 MGUSs, 12 SMMs, 3792 MMs, and 30 PCLs. The datasets of GSE39754 (*n* = 176), GSE5900 (*n* = 78), GSE116294 (*n* = 69), GSE13591 (*n* = 162), and GSE2113 (*n* = 52) were used for microarray expression analysis; GSE24080 (*n* = 559), GSE4204 (*n* = 538), GSE31161 (*n* = 1038), and GSE83503 (*n* = 602) were analyzed to determine the relationship between SRSF1 expression and age, ISS staging, 1q21 abnormalities, or disease relapse. For survival analysis, GSE24080 (*n* = 559), GSE4204 (*n* = 538), GSE2658 (*n* = 599), and GSE57317 (*n* = 55) were used. Among these datasets, GSE31161 and GSE83503 contain 255 and 403 relapsed patients, respectively; while the remaining datasets include only patients with newly diagnosed MM. The cancer dependency score of SRSF1 was acquired from the DEPMAP portal. DEPMAP portal is a genome-wide CRISPR screening database that identifies essential genes for tumorigenesis (https://depmap.org/portal) [25]. A lower score means a gene is more likely to be dependent in a given tumor cell line. A median score of 0 means a gene is not essential for tumor cells, whereas a median score of −1 is equivalent to a gene that is essential for tumor cell lines.

### 2.2. Exploring the Role of SRSF1 in MM

The protein-protein interaction (PPI) network of the SRSF family was analyzed through the Search Tool for Interaction Genes (STRING) database [26]. Pearson's correlation test was applied to evaluate the relationship between members of the SRSF family. To define the prognostic role of the SRSF family, univariate Cox regression was conducted in GSE24080. The expression of the SRSF family between healthy donors and MM patients was analyzed in the GSE39754 dataset, and SRSF1 was chosen to do further analysis due to its differential expression and prognostic value. The relationship between SRSF1 expression and the clinical characteristics of MM patients was analyzed in GSE24080. Patients were divided into the SRSF1^low^ group and the SRSF1^high^ group based on the median expression values of SRSF1. Kaplan–Meier methods and log-rank test were used for survival analysis. Univariate Cox regression and multivariate Cox regression were constructed for event-free survival (EFS) and overall survival (OS), using the “Backward: LR” procedure. The confidence interval (CI) was 95%.

### 2.3. Identification of Differentially Expressed Genes and Enrichment Analysis

Differential gene expression analysis was performed by the “limma” package [27]. |Fold change | > 1.5, and *p* < 0.05 was utilized to determine differentially expressed genes (DEGs). Gene Ontology (GO) enrichment terms and Kyoto Encyclopedia of Genes and Genomes (KEGG) pathways were performed by “clusterProfiler” package [28]. Gene set enrichment analysis (GSEA) was performed by GSEA software (https://www.gsea-msigdb.org/gsea/index.jsp) [29].

### 2.4. Analysis of Immune Cell Characteristics and Immune-Specific Gene Expression between SRSF1^high^ and SRSF1^low^ Groups

Immune cell characteristics between the SRSF1^high^ and SRSF1^low^ groups were analyzed by ImmuCellAI (Immune Cell Abundance Identifier), an online tool to provide the quantitative infiltration of immune cells by using gene expression matrix data (http://bioinfo.life.hust.edu.cn/ImmuCellAI) [30]. ESTIMATE was used to score the tumor microenvironment (TME) of samples, including stromal score, immune score, ESTIMATE score, and tumor purity [31]. Differences in the TME between the SRSF1^high^ and SRSF1^low^ groups were analyzed. The correlation between SRSF1 expression and immune cell infiltration was calculated by Pearson correlation analysis.

### 2.5. Cell Culture and Primary Cells from Normal Donors and MM Patients

KM3, U266, 8226, and H929 cells were cultured in Roswell Park Memorial Institute (RPMI)-1640 medium (Gibco, USA) with 10% fetal bovine serum (FBS, Gibco, USA), 100 *μ*/mL penicillin, and 100 mg/mL streptomycin. All cells were acquired from the American Type Culture Collection (ATCC, USA). Eight patients with MM and three patients with benign diseases were included, and informed consents were obtained from all the participants. The mononuclear cells were isolated from the bone marrow samples using Ficoll density centrifugation.

### 2.6. Reverse Transcription-Quantitative Polymerase Chain Reaction (RT-qPCR) and Western Blotting

Total RNA was extracted from mononuclear cells by TRIZOL reagent (Invitrogen, USA) and was then reverse transcribed to cDNA using PrimeScript™ RT reagent Kit (Takara, Japan) according to the manufacturer's instruction. Real-time fluorescent quantitative PCR was performed to amplify the SRSF1 cDNA fragment by SYBR Green Master Mix (Yeasen, China), with *β*-actin as an internal control. The expression level of related lncRNAs was analyzed using 2^−ΔΔCT^. Each PCR reaction was performed in triplicate. The following primers were used: SRSF1 forward, 5′-GCCGCATCTACGTGGGTAAC-3′; SRSF1 reverse, 5′-GAGGTCGATGTCGCGGATAG-3′;*β*-actin forward, 5′-GATCATTGCTCCTCCTGAGC-3′;*β*-actin reverse, 5′- ACTCCTGCTTGCTGATCCAC-3′. The expression of SRSF1 in MM cell lines was detected by western blotting as previously described [32]. Primary rabbit anti-SRSF1 antibody was purchased from Abcam (ab133689), and primary mouse anti-*β*-tubulin antibody was purchased from HUABIO (M1305-2).

### 2.7. Knockdown of SRSF1 and Cell Proliferation Assay

We used short hairpin RNA (shRNA) to reduce SRSF1 expression. The shSRSF1 and shctrl plasmids were constructed with the PLKO.1 vector (Addgene, US). shSRSF1 target sequence: GCTGATGTTTACCGAGATGGC; control target sequence: TTCTCCGAACGTGTCACGT. The target and control plasmids were separately cotransfected with the lentiviral packaging plasmids (pM2D.G and psPAX2) into HEK293 T cells with Liposomal Transfection Reagent (Yeasen, China) to produce lentiviruses. Target cells were infected with the virus and 10 *μ*g/ml polybrene (Sigma, US) for 24 hours, and 2 *μ*g/ml puromycin was added at 72 hours after infection. For the cell proliferation assay, a total of 5 × 10^^3^ H929 and 2 × 10^^3^ U266 were seeded in 96-well plates in triplicates and cultured at 37°C. Cell proliferation was determined by CCK-8 assays (Dojindo Lot.JE603).

### 2.8. Statistical Analysis

SPSS statistical software (SPSS statistics 23.0), R software (version 3.6.3), GraphPad Prism 8.0, and GSEA software were used for statistical analyses. Gene expression datasets were obtained by using Affymetrix Human Genome 133 plus 2.0 Array. All experiment design, quality control, and data normalization follow the standard Affymetrix protocols. This study was conducted in accordance with the International Conference and the Declaration of Helsinki. Each dataset was first evaluated for normality of distribution by the Kolmogorov–Smirnov test to decide whether a nonparametric rank-based analysis or a parametric analysis should be used. The Fisher exact and Wilcoxon rank-sum tests were used to compare categorical and numerical data, respectively.

## 3. Results

### 3.1. The Role of SRSF Family in MM

**Figure 1:**
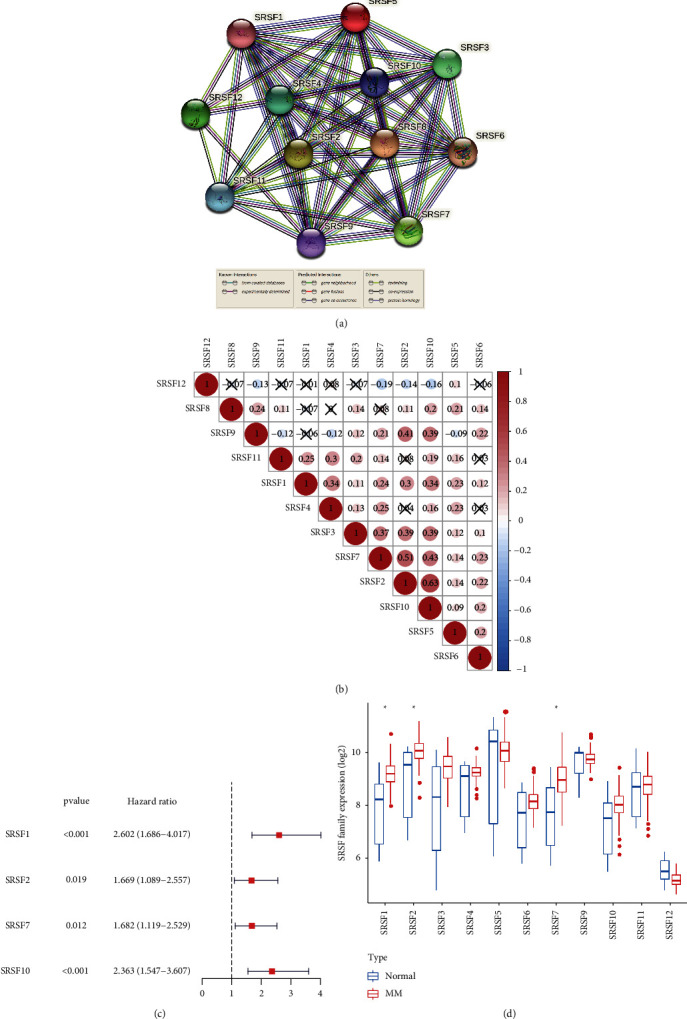
Analysis of SRSF family in multiple myeloma patients. (a) The PPI network of SRSF family members. (b) Correlation analysis between the expression of the SRSF family in the GSE24080 dataset. The size of the dot represents the correlation coefficient, and the larger the dot, the higher the correlation. Red dots represent a positive correlation, while blue dots represent a negative correlation. (c) Univariate Cox regression results for the subunits related to the survival of MM patients in GSE24080. (d) The expression of SRSF family members between normal donors and MM patients in GSE39754.

To explore the potential role of the SRSF family in MM, we performed gene expression profiling in GSE24080. The STRING database showed a close PPI network among SRSF family genes (Figure 1(a)). The RNA expression levels were correlated with each other among SRSF members, and the correlation was the strongest between SRSF2 and SRSF10 (*r* = 0.67, Figure 1(b)). We next investigated whether SRSF members are prognostic for MM through univariate Cox regression analysis, and SRSF1, SRSF2, SRSF7, and SRSF10 were found to be associated with OS of MM patients (Figure 1(c), all *p* < 0.05, all hazard ratio (HR) > 1). Moreover, we utilized the GSE39754 dataset to screen differentially expressed SRSF genes between healthy donors and MM patients. As shown in Figure 1(d), SRSF1, SRSF2, and SRSF7 were expressed significantly higher in MM than those in healthy control, and among these, SRSF1 was top-ranked in terms of HR value and was selected for further analysis.

### 3.2. The Expression Level of SRSF1 in Normal Donors and Multiple Myeloma Patients in Different Stages

**Figure 2:**
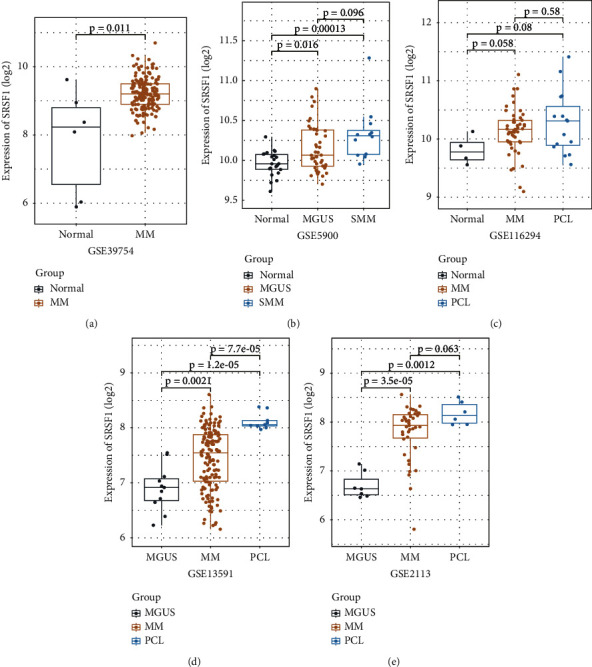
The expression level of SRSF1 in five datasets of normal donors and myeloma patients in different stages: (a) MM patients (*n* = 170) compared with normal donors (*n* = 6); (b) the expression value of SRSF1 in normal donors (*n* = 22) and other different stages of 56 myeloma patients. MGUS (*n* = 44) and SMM (*n* = 12); (c) the different expressions of SRSF1 in normal donors (n = 4), MM [33], and PCL patients (*n* = 15); (d) SRSF1 expression levels in different subtypes of myeloma patients. MGUS (*n* = 11), MM (*n* = 133), and PCL (*n* = 9); (e) comparison of SRSF1 expression levels in three different stages of myeloma patients. MGUS (*n* = 7), MM (*n* = 39), and PCL (*n* = 6).

To characterize the SRSF1 expression pattern related to MM development, we employed five datasets to determine the SRSF1 mRNA levels in different stages of MM, including MGUS, SMM, MM, and PCL. In GSE39754, the expression level of SRSF1 was significantly higher in 170 MM patients than that in 6 normal donors (*p*=0.011) (Figure 2(a)). A prominent trend of increase in SRSF1 expression was found from normal control (*n* = 22), MGUS (*n* = 44) to SMM (*n* = 12) in GSE5900 (*p*=0.016, 0.00013, and 0.096, respectively, Figure 2(b)). Similarly, the GSE116249 dataset showed that SRSF1 expression increased from normal control (*n* = 4), MM (*n* = 50) to PCL (*n* = 15), even though the difference is not statistically significant (*p*=0.058, 0.08, and 0.58, respectively, Figure 2(c)). Moreover, the expression of SRSF1 significantly increased in MGUS (*n* = 11), MM (*n* = 142), and PCL (*n* = 9) (*p*=0.0021, 1.2*e* − 05, and 7.7*e* − 05, respectively, Figure 2(d)). The same trend was also found in the GSE2113 dataset, from MGUS (*n* = 7) to MM (*n* = 39) and PCL (*n* = 6) (*p*=3.5*e* − 05, 0.0012, and 0.063, respectively, Figure 2(e)). Together, these data suggest that SRSF1 was overexpressed during the courses of MM progression.

### 3.3. The Expression Level of SRSF1 in MM Patients with Different Age Groups, ISS Stages, Amplification of 1q21, and Relapse Statuses

**Figure 3:**
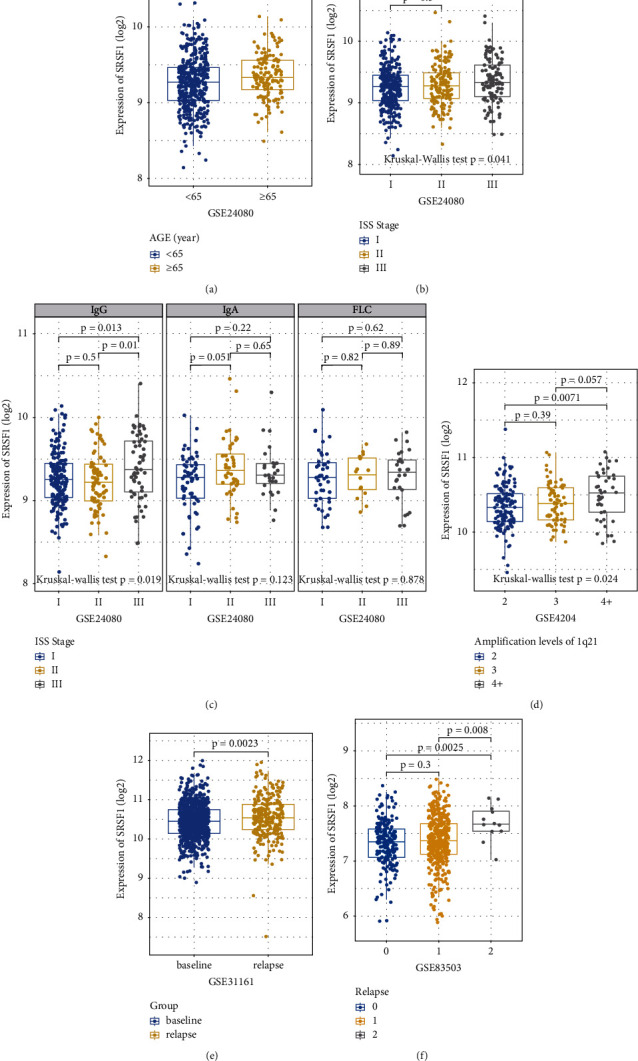
The expression of SRSF1 in different age groups, ISS stages, amplification levels of 1q21, and relapse status of MM patients: (a) the expression level of SRSF1 in ages <65 years old (*n* = 423) and ≥65 years old (*n* = 136) groups, (b) the expression of SRSF1 in different ISS stages, (c) the SRSF1 expression pattern in different serotypes, (d) SRSF1 expression levels at different 1q21 amplification in 246 MM patients of GSE4204, (e) the expression of SRSF1 in baseline (*n* = 780) and relapse (*n* = 255) MM groups, and (f) SRSF1 expression levels in 585 MM patients with different relapse times: relapse time = 0 (*n* = 182), relapse time = 1 (*n* = 391), and relapse time = 2 (*n* = 12).

To better understand the clinical characteristics associated with SRSF1 expression, MM patients' age, ISS stages, 1q21 aberrations, and relapse status were analyzed using four independent datasets. In GSE24080, SRSF1 expression in the group of age ≥65 was significantly higher than that in the group of age <65 (*p* = 0.0056, Figure 3(a)). Furthermore, a significantly higher SRSF1 expression was observed in ISS stage III when compared with stage I or II (*p* = 0.011, Kruskal–Wallis test, Figure 3(b)), which specifically occurs in IgG-type MM (*p* = 0.013 and 0.01, respectively, Kruskal–Wallis test, Figure 3(c)), but not in free light chain (FLC)- or IgA-type MM (*p* = 0.878 and 0.123, respectively, Figure 3(c)).

The 1q21 copy number amplification is a common cytogenetic abnormality that is indicative of poor prognosis in patients with MM [33–35]. In GSE4204 (*n* = 538), the expression of SRSF1 had an upward trend with the amplification of 1q21 (*p* = 0.024, Kruskal–Wallis test, Figure 3(d)), and a similar result was obtained in GSE2658 (*n* = 599) (Supplementary Figure 1). Furthermore, in GSE31161, we found a significant increase of SRSF1 expression in relapsed MM patients (*n* = 258) when compared with MM patients at diagnosis (*n* = 780) (*p* = 0.00028, Figure 3(e)). Besides, we found that SRSF1 expression increased with the duration of relapse in the GSE83530 dataset (*p* = 0.3, 0.008, and 0.0025, respectively, Figure 3(f)), suggesting that SRSF1 may contribute to the relapse of MM patients.

### 3.4. Clinical and Molecular Characteristics of Patients between SRSF1^high^ and SRSF1^low^ Groups

Using the GSE24080 dataset, we divided 559 patients into two groups, including the SRSF1^low^ (*n* = 280) and the SRSF1^high^ (*n* = 279) groups. Then, we analyzed the clinical and molecular characteristics between the two groups (Table 1). Compared with the SRSF1^low^ group, the SRSF1^high^ group was more likely related to race (*p*=0.003), advanced ISS stage (*p*=0.047), and increased beta-2 microglobulin (B2M) (*p*=0.005). The incidence of cytogenetic abnormality was higher in the SRSF1^high^ group than in the SRSF1^low^ group, although there was no significant statistical difference (*p*=0.09). Additionally, the SRSF1^high^ group was associated with high expression of NOTCH2NL, MYBL2, and UBE2T and low expression of CXCR4 and IL18R1 (all *p* < 0.05). NOTCH2NL, MYBL2, and UBE2T were reported to be involved in tumorigenesis [36–38], while CXCR4 and IL18R1 were associated with immune response and pathway activation [39, 40], indicating SRSF1 may take part in MM progression via tumor-related pathways. Between the two groups, there were no significant differences in age, gender, isotype, and therapy options.

### 3.5. Univariate and Multivariate Analysis of Possible Prognostic Factors in MM

To further evaluate the potential prognostic value of SRSF1 in MM, age (≥65 vs. <65), gender (female vs. male), B2M (≥5.5 vs. <5.5), LDH (≥250 vs. <250), ALB (≥3.5 vs. <3.5), and lytic bone lesions on MRI (≥2 vs. <2) were enrolled in univariate and multivariate analysis. As a result, SRSF1, B2M, and LDH were significantly associated with EFS in univariate analysis (all *p* < 0.05) (Table 2). Furthermore, the multivariate analysis showed that HR values of SRSF1, B2M (≥5.5 vs. 5.5), and LDH (≥250 vs. <250) were 1.851 (*p* < 0.001), 1.614 (*p*=0.008), and 2.590 (*p* < 0.001), respectively. Additionally, SRSF1, B2M, LDH, ALB, and MRI lesions were identified to be closely related to OS in univariate analysis (all *p* < 0.05) (Table 3). Furthermore, the multivariate analysis for OS displayed that the HR of SRSF1 was 1.720 (*p*=0.001), and the HR values of other OS-related factors, including B2M, LDH, ALB, and MRI, were 1.922, 2.909, 0.656, and 1.737 (*p* < 0.001, <0.001, 0.034, and <0.002, respectively). These results suggested that SRSF1 expression was an independent risk factor affecting the survival of MM patients.

### 3.6. SRSF1 Predicted Survival Levels in MM Patients

**Figure 4:**
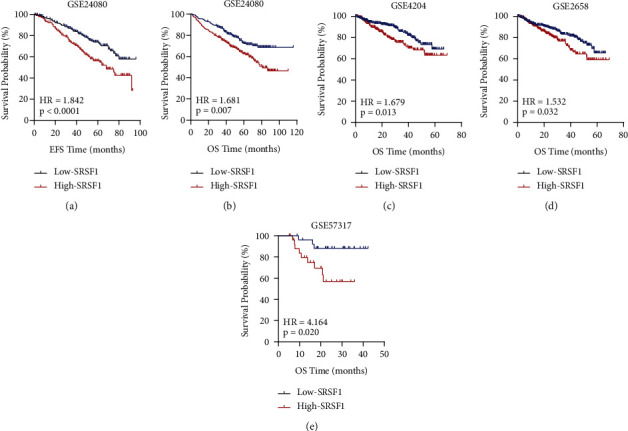
Survival analysis of the SRSF1^high^ and SRSF1^low^ groups. The *X*-axis represents the survival time (month), and the *Y*-axis represents survival probability. (a, b) Analysis of EFS and OS between the SRSF1^high^ and SRSF1^low^ groups in GSE24080 (*n* = 559). (c) OS between SRSF1^high^ and SRSF1^low^ in GSE4204 (*n* = 538). (d) OS analysis of 559 pretreatment MM patients in the GSE2658 dataset. (e) OS analysis of MM patients after treatment in GSE57315 (*n* = 55).

To validate the poor prognosis conferred by high SRFS1 expression in MM patients, we conducted survival analysis in GSE24080 and other independent cohorts. In GSE24080, we found that the SRSF1^high^ group had significantly shorter EFS and OS than the SRSF1^low^ group, both *p* < 0.001 (Figures 4(a) and 4(b)). A similar prognostic value of SRSF1 was demonstrated in GSE4204, GSE2658, and GSE57317 (*p*=0.013, 0.032, and 0.020, respectively, Figures 4(c)–4(e)). Since B2M and LDH are recognized as important prognostic biomarkers for MM, we further explored the prognosis of SRSF1 expression levels in the B2M and LDH subgroups of GSE24080. In the B2M ≤ 3.5 mg/l and LDH < 250 U/L groups, the SRSF1^high^ group had significantly shorter EFS and OS than the SRSF1^low^ group (EFS: *p* < 0.001 and *p* < 0.001; OS: *p*=0.0026 and *p*=0.001, respectively. Supplementary Figures 2A and 2D; Supplementary Figures 3A and 3C). In the LDH ≥250 U/L group, patients with high SRSF1 expression tended to have shorter EFS and OS than those with low SRSF1 expression, even though these differences were not statistically significant (EFS: *p*=0.073 and OS: *p*=0.113, respectively. Supplementary Figures 3B and 3D). While in the 3.5 < B2M < 5.5 mg/l and B2M ≥ 5.5 mg/l groups, there were no significant differences in OS and EFS between the SRSF1^high^ and the SRSF1^low^ expression groups (Supplementary Figures 2B–2F). The lack of difference may suggest that the deleterious impact of B2M ≥ 5.5 mg/l on prognosis may override that of high SRSF1 expression.

### 3.7. Differential Gene Expression and Pathway Enrichment Analysis for SRSF1^high^ versus SRSF1^low^

**Figure 5:**
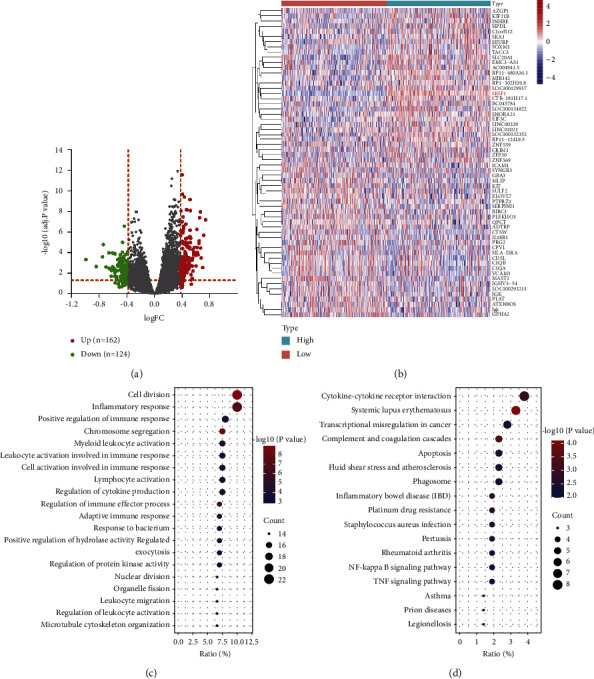
Differently expressed genes (DEGs) and the results of GO and KEGG enrichment analysis. (a) Volcano plot of the DEGs expression between the SRSF1^high^ and SRSF1^low^ groups. Green dots represent 126 downregulated genes, red dots represent 163 upregulated genes, and grey dots indicate nonsignificant genes. (b) Heatmap shows top 30 upregulated genes and the top 30 downregulated genes. Red represents high expression, white represents intermediate expression, and blue represents low expression. (c-d) Top 20 terms of GO and KEGG enrichment analysis for differential expressed genes.

To gain insight into SRSF1 biological functions, we tried to identify the DEGs by comparing the SRSF1^high^ group with the SRSF1^low^ group in GSE24080. A total of 289 DEGs related to SRSF1 were identified, of which 162 were upregulated and 124 were downregulated (*p* < 0.05, |log2 FC|≥0.378, Figure 5(a), Supplementary Table 1). The heatmap showed the top 30 upregulated genes and 30 downregulated genes (Figure 5(b)). Furthermore, we analyzed the top 20 GO terms and KEGG pathways to identify enriched categories and signaling pathways (Supplementary Tables 2 and 3). DEGs were mainly enriched in cell division, inflammatory response, and positive regulation of immune response (Figure 5(c)). In the KEGG pathway analysis, cytokine-cytokine receptor interaction, systemic lupus erythematosus, transcriptional misregulation in cancer, and complement and coagulation cascades were the most enriched pathways (Figure 5(d)).

### 3.8. The PPI Network and Correlation Analysis of DEGs

**Figure 6:**
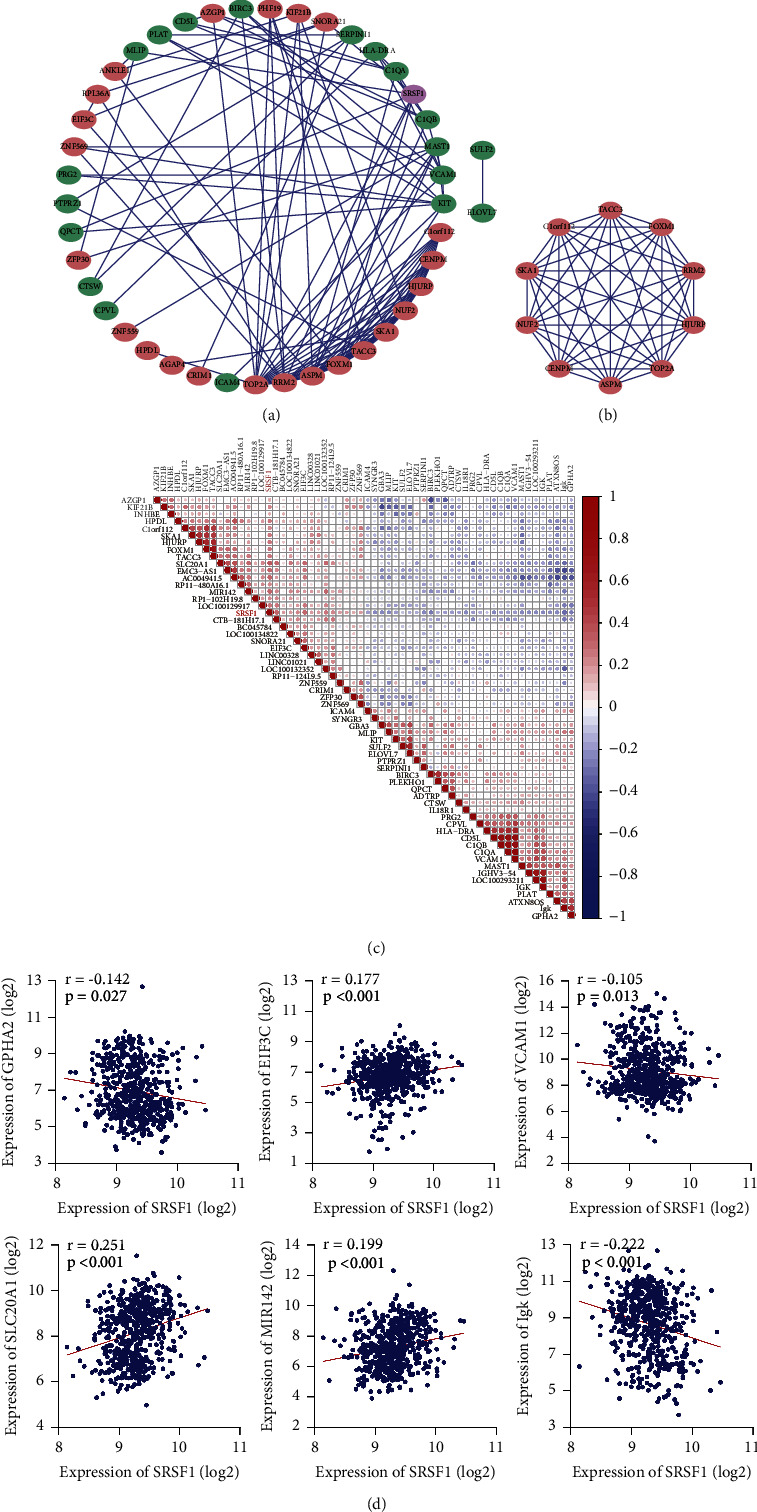
The PPI and correlation analysis of DEGs. (a) PPI network of top 60 DEGs. (b) The core subnetwork of the PPI network by using MCODE APP in Cytoscape. (c) The correlation analysis of DEGs with the Pearson correlation coefficient. The red circle means a positive correlation, while the blue circle means a negative correlation. (d) The correlation analysis of SRSF1 and DEGs.

The PPI network in the STRING database showed the top 60 SRSF1-related DEGs' interaction (Figure 6(a)). Then, we discovered the subnetwork by using the MCODE in the Cytoscape (Figure 6(b)). In addition, we used the top of 60 DEGs to calculate the correlativity between those genes. Based on the expression heatmap, we found that SRSF1 was positively correlated with SLC20A1, MIR142, and EIF3C and negatively associated with IgK, GPHA2, and VCAM1 (Figure 6(c), all *p* < 0.001). In addition, there were positive correlations between SRSF1 and many noncoding RNAs, such as EMC-AS1 and MIR142, and the function of most noncoding RNA in MM is still unknown.

### 3.9. GSEA Analysis Showed a Lot of Gene Sets Enriched in the SRSF1^high^ Group

**Figure 7:**
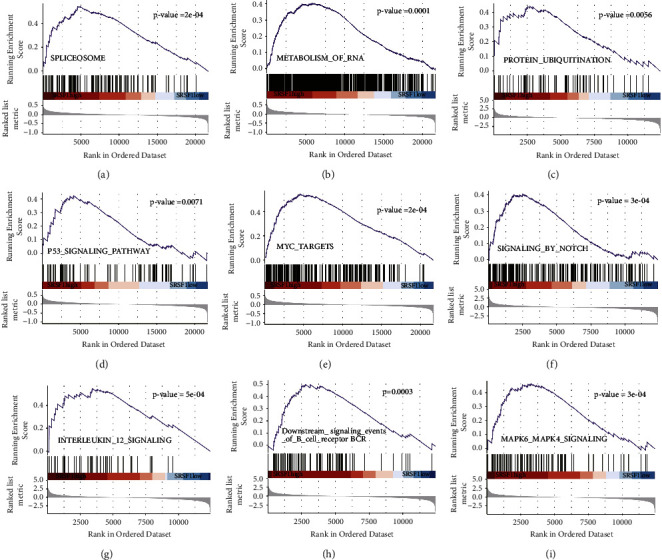
Enrichment analysis of gene signaling pathways between the SRSF1^high^ group and the SRSF1^low^ groups. GSEA showed that spliceosome (a), metabolism of RNA (b), protein ubiquitination (c), P53 signaling pathway (d), MYC targets (e), signaling by NOTCH (f), interleukin 12 signaling (g), downstream signaling events of B cell receptor BCR (h), and MARK6/4 signaling (i) were enriched in the SRSF1^high^ group. All *p* < 0.01.

The GSEA analysis showed that spliceosome, metabolism of RNA, protein ubiquitination, P53 signaling pathway, MYC targets, signaling by NOTCH, interleukin 12 signaling, downstream signaling events of B cell receptor BCR, and MARK6/4 signaling were significantly enriched in the SRSF1^high^ group (Figures 7(a)–7(i), all *p* < 0.01), while hematopoietic cell lineage, B cell receptor signaling pathway, TNFa signaling via NFKB, inflammatory response, complement, and coagulation were significantly enriched in the SRSF1^low^ group (Supplementary Figure 4, all *p* < 0.01).

### 3.10. Analysis of Immune Cell Characteristics and Immune-Specific Gene Expression between the SRSF1^high^ and SRSF1^low^ Groups

**Figure 8:**
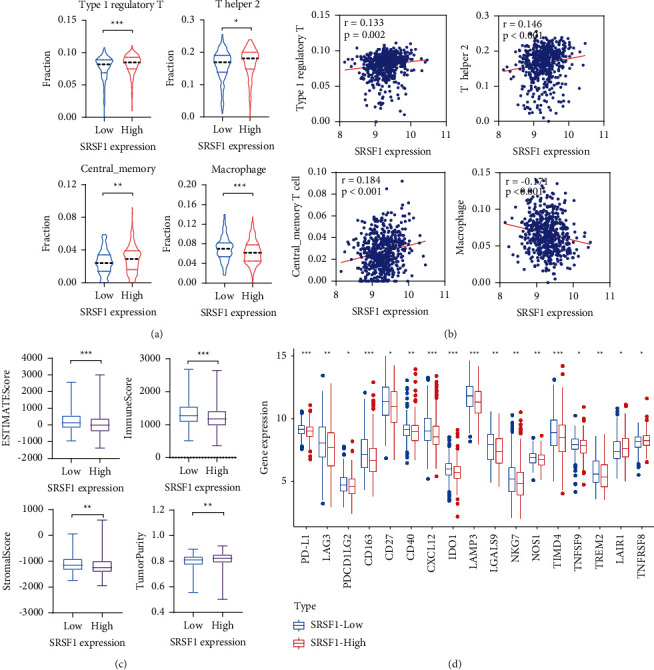
Analysis of tumor-infiltrating immune cells and immune-related genes in the SRSF1^high^ and SRSF1^low^ groups: (a) the fraction of 4 types of tumor immune infiltrating cells in two groups, (b) correlation of SRSF1 expression with 4 tumor-infiltrating immune cell subtypes, (c) tumor microenvironment characteristics in the SRSF1^low^ and SRSF1^high^ groups, and (d) the expression levels of immune-related genes between the SRSF1^high^ and SRSF1^low^ groups. ^*∗*^*p* < 0.05, ^*∗∗*^*p* < 0.01, and ^*∗∗∗*^*p* < 0.001.

ImmuCellAI is an online tool to estimate the infiltration of immune cells based on the gene expression matrix data [30]. The abundance of immune cells with significant differences between the high- and low-risk groups is shown in Figure 8(a). The levels of type 1 regulatory T (Tr1) cells, T helper 2 (Th2) cells, and central memory T (TCM) cells in the SRSF1^high^ group were higher than those in the SRSF1^low^ group, while the levels of macrophage cells in the SRSF1^high^ group were lower than that in the SRSF1^low^ group (all *p* < 0.05). To determine the correlation between SRSF1 expression and tumor-infiltrating immune cells, we found that the expression level of SRSF1 was positively correlated with Tr1, Th2, and central memory T cells and negatively correlated with macrophage cells (Figure 8(b), all *p* < 0.05). We also used the ESTIMATE algorithm to evaluate the TME between the two groups. Notably, patients with a high SRSF1 expression presented a lower ESTIMATE score, immune score, and stromal score (all *p* < 0.05) and a higher level of tumor purity(*p* < 0.05) (Figure 8(c)). We observed that immune checkpoint markers, consisting of PD-L1, LAG3, and PDCD1LG2, were remarkably downregulated in the SRSF1^high^ group. Moreover, genes associated with immune response activation, including CD163, CD27, CD40, CXCL12, IDO1, LAMP3, LGALS9, NKG7, NOS1, TIMD4, TNFSF9, and TREM2, were downregulated in SRSF1 high expression MM patients, while genes related to immune response limitation such as LAIR1 and TNFRSF8 were upregulated in SRSF1 low expression MM patients. Taken together, these data suggested that SRSF1 was related to tumor immune infiltrating cells and may have participated in tumor immune escape in MM.

### 3.11. Validation of the Expression and Function of SRSF1 in MM

**Figure 9:**
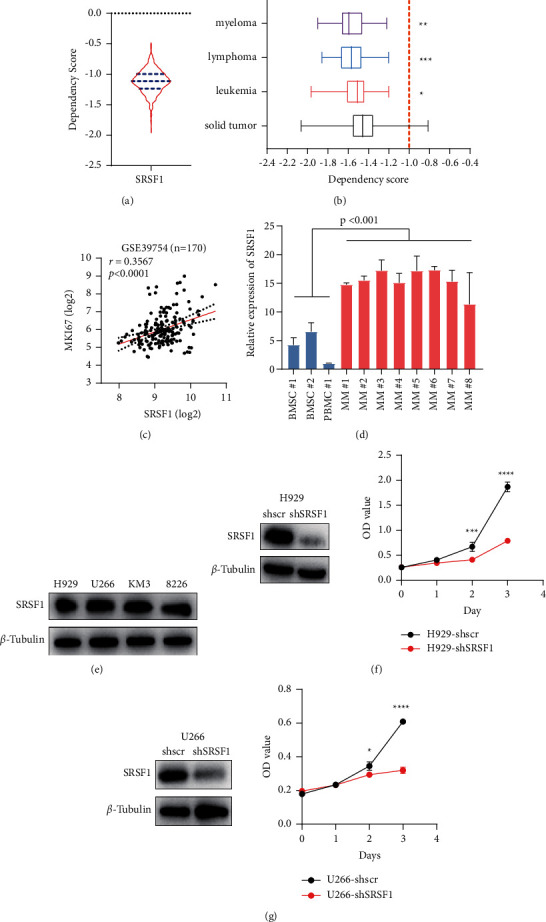
Expression of SRSF1 in MM patients and SRSF1 is essential for MM cell proliferation. (a) The dependency score of SRSF1 in tumor cell lines. A lower score means a gene is more likely to be dependent in a given cell line. A median score of 0 means a gene that is not essential, whereas a median score of −1 means a gene that is a common essential gene for tumor cell lines. (b) SRSF1 dependency score between hematological malignancies and solid tumors. ^*∗*^*p* < 0.05, ^*∗∗*^*p* < 0.01, and ^*∗∗∗*^*p* < 0.001. (c) Correlation of SRSF1 expression and MKI67 expression in GSE39754 (*n* = 170). (d) The expression level of SRSF1 between three normal samples and eight MM patients. (e) The expression of level of SRSF1 in four MM cell lines. (f, g) SRSF1 knockdown inhibits the growth of MM cell lines: H929 and U266.

In order to test whether SRSF1 is dispensable for the survival of cancer cells, we extracted the cancer dependency score of SRSF1 from a genome-wide CRISPR screening database, the DEPMAP portal. A lower score means that a gene is more likely to be dependent in a given tumor cell line. A median score of 0 means that a gene is not essential for tumor cells, whereas a median score of −1 is equivalent to a gene that is essential for tumor cell lines. The dependency score of SRSF1 is −1.113, which means that SRSF1 is a common essential gene for tumor cell lines (Figure 9(a)). Across more than 500 lines representing 28 different cancer cell lineages, the dependency scores of SRSF1 in hematological malignancies, especially myeloma, were significantly lower than those in solid tumors, suggesting SRSF1 might play an essential role in hematological malignancies (Figure 9(b)). Additionally, the expression level of SRSF1 had a positive correlation with MKI67 expression in MM patients (Figure 9(c), *r* = 0.3567, and *p* < 0.0001), indicating the role of SRSF1 in myeloma development.

To validate our findings, we first tested the expression level of SRSF1 in a cohort of three control donors and eight MM patients using RT-qPCR analysis (Figure 9(d)). The result showed that SRSF1 expression was significantly higher in MM patients than that in normal honors. Western blot showed that SRSF1 was commonly expressed in MM cell lines (Figure 9(e)). To investigate the role of SRSF1 in MM cell proliferation, we used short hairpin RNA (shRNA) to reduce SRSF1 expression in H929 and U266 cell lines. The proliferation assay showed that SRSF1 knockdown significantly inhibited the growth of H929 and U266 (Figures 9(f)–9(g)). These results were consistent with our previous findings, indicating that SRSF1 plays an essential role in the development and progression of multiple myeloma.

## 4. Discussion

In recent years, treatments for MM patients have achieved significant advances, while drug resistance is still a critical feature of the disease and contributes to disease relapses and poor overall survival. Thus, finding predictive biomarkers is essential for improving treatment results in MM patients.

In this study, we acquired RNA expression data and clinical information of MM patients from the GEO database. Firstly, we investigated the role of SRSF family members in MM and screened SRSF1 as the most potential factor for further analysis. SRSF1 was upregulated in newly diagnosed and relapsed MM patients. Furthermore, SRSF1 overexpression was associated with several adverse clinical parameters, including old age, high levels of B2M and LDH, low ALB level, and 1q21 amplification. Univariate and multivariate Cox regression was conducted to identify whether SRSF1 was an independent prognostic factor for MM. Survival analysis revealed that patients in the SRSF1^high^ group had a much worse prognosis than those in the SRSF1^low^ group. These results turned out that the SRSF1 expression level can be used as a potential predictor in MM prognosis.

The high expression level of SRSF1 has been shown to confer poor prognosis in a variety of cancers, and the underlying mechanisms were characterized. For example, SRSF1 overexpression was reported to increase tumor invasion and metastasis in hepatocellular carcinoma [18]. Du et al. suggested that SRSF1 promotes the progression of breast cancer through oncogenic splice switching of PTPMT1 [19]. Additionally, SRSF1 involves in both normal and malignant hematopoiesis. To protect T cell intrathymic maturation, SRSF1 regulates various cellular processes, such as cell differentiation, proliferation, apoptosis, and type I interferon signaling pathway [21]. NSrp70, a splicing factor, regulates thymocyte development via partial alternative processing of SRSF1 [22]. Sinnakannu et al. reported that high SRSF1 expression in chronic myeloid leukemia was associated with imatinib resistance, which was mediated by the SRSF1/PRKCH/PLCH1 axis [24]. In AML, SRSF1 was responsible for the generation of alternative isoforms of proapoptotic and antiapoptotic genes, including BCL-x, MCLs, and capsase9b [41]. In pediatric acute lymphoblastic leukemia (ALL), SRSF1 was upregulated in clinical samples from de novo or relapsed patients and decreased when complete remission was achieved [23]. In our study, we also found that the expression level of SRSF1 was significantly higher in the relapsed MM compared with newly diagnosed MM and increased with disease recurrences. Alternative splicing events can produce tumor-related splice variants and proteins [42]. Thus, it is tempting to investigate whether increased SRSF1 expression affects alternative splicing to drive MM progression.

To explore the potential biological mechanism of SRSF1 overexpression, GO, KEGG, and GSEA analyses were performed. It turned out that pathways were enriched in cell division, inflammatory response, cytokine-cytokine receptor interaction, RNA metabolism, and transcriptional misregulation, specifically P53, MAPK4/6, NOTCH, and MYC pathways, which are critical regulators involved in myeloma initiation and progression. SRSF1 is important for spliceosome formation and RNA metabolism, and dysregulation of RNA stability could promote MM progression [43]. Moreover, SRSF1 has been identified as an MYC-sensitive oncogenic protein [13], suggesting that abnormal SRSF1 expression might affect MYC-related pathways. It has been established that p53, NOTCH, and MAPK6/4 signaling pathways play important roles in MM initiation and progression [44, 45]. Moreover, IL-12 and B cell receptor (BCR) signaling are essential for an immune response [46–48]. Therefore, it is necessary to further investigate whether SRSF1 promotes MM development through immune modulation.

Bone marrow microenvironment (BMME) is important for MM initiation and progression. Components of BMME, such as immune effector cells and immune molecules, can be abnormally edited, which further promote MM progression by enhancing initial immunotolerance and subsequent tumor cell escape from immune surveillance [49]. We found that Tr1, Th2, and TCM exhibited a higher degree of infiltration in the SRSF1^high^ group, while the degree of macrophage infiltration was higher in the SRSF1^low^ group. Correlation analysis showed that the SRSF1 expression level was positively correlated with Tr1, Th2, and TCM and negatively associated with macrophages. Tr1 cells play a role in inflammatory responses and immune tolerance; however, dysfunction of Tr1 cells may limit antitumor immunity [50, 51]. Studies have shown that Th2 cells are closely associated with MM progression. Increased Th2 cells lead to a closer myeloma cell interaction, which subsequently contributes to MM development [52–55]. Recently, immune checkpoint therapy has achieved great breakthroughs in the treatment of hematological malignancies. Finding reliable biomarkers and potential targets can provide new sights for immunotherapy in MM. Therefore, we compared checkpoint markers and immune-related genes between the SRSF1^high^- and SRSF1^low^ groups. We observed that checkpoint markers such as PD-L1, LAG3, and PDCD1LG2 were downregulated in the SRSF1^high^ group. PD-L1 inhibitors, such as durvalumab and pembrolizumab, have been reported to be effective in the treatment of relapsed or refractory MM [56–58]. SRSF1 expression might provide a new idea for immune checkpoint inhibitor therapy. In MM patients with high SRSF1 expression, immune-related genes for immune response activation were remarkably downregulated, whereas immunosuppressive genes were increased, indicating SRSF1 might play a role in modulating the expression of immune-related genes. Additionally, alternative processing of mRNA has been reported to have the potential to provide new therapeutic targets for cancer immunotherapy [59]. Thus, splicing variants and proteins produced by alternative splicing caused by abnormal expression of SRSF1 may provide a new insight for immunotherapy in MM patients. Altogether, our findings showed that the SRSF1 expression level could affect tumor immune characteristics via infiltrating immune cells, TME, checkpoint markers, and immune-related genes, thereby determining the prognosis of patients with MM.

SRSF1 has been widely reported as an oncogene in many tumors. We identified SRSF1 as an essential cancer-dependent gene in tumorigenesis by using the DepMap database, especially in multiple myeloma. To validate the bioinformatic results, first, we performed RT-qPCR on clinical samples and found that SRSF1 expression was significantly increased in MM patients compared with controls. Then, we found that the knockdown of SRSF1 led to growth inhibition of MM cell lines. Combined with Figures 3 and5, patients with the high expression level of SRSF1 were associated with worse outcomes, indicating that SRSF1 can be a promising biomarker and target in MM diagnosis and treatment.

Although we briefly profiled the SRSF1-induced gene expression, this study has some limitations. Firstly, whether or how SRSF1 affects the splicing events of target genes in MM needs to be explored. Secondly, the number of MM patients enrolled for validation was small. We are collecting more primary MM samples to further detect and correlate SRSF1 expression with the clinical outcomes of MM patients. In our study, we identified the SRSF1 expression in MM with different ages, ISS stages, amplification of 1q21, and relapse statuses in newly diagnosed and relapsed MM patients. Since MM is a heterogeneous disease with different patterns of clonal evolution [60], to better evaluate SRSF1 as an important prognostic factor, MM patients with various genetic alterations and disease statuses should be enrolled in the future study.

## 5. Conclusion

In conclusion, our study revealed that SRSF1 expression is upregulated in MM patients. Notably, SRSF1 overexpression was associated with worse clinical characteristics in MM patients (age, ISS stage, amplification of 1q21, relapse statuses as well as beta-2 microglobulin) and predicts poor OS and EFS of MM patients. Additionally, the knockdown of SRSF1 repressed the growth of MM cell lines. Thus, our results demonstrated that SRSF1 may promote MM growth with prognostic significance and can potentially be used as a novel biomarker in the future. Moreover, more research studies need to be carried out to explore the complicated mechanisms of SRSF1 in MM development and progression.

## Figures and Tables

**Table 1:** Clinical characteristics of 559 MM patients between the SRSF1^low^ and SRSF1^high^ groups in GSE24080.

		SRSF1^Low^, *n* = 280	SRSF1^High^, *n* = 279	*p* value
Age (mean, range)		57.74 (24.83–75.90)	56.62 (31.82–76.50)	0.161
Gender (%)	Male	175 (62.50)	162 (58.06)	0.284
	Female	105 (37.50)	117 (41.94)	
Race (%)	White	238 (85.00)	259 (92.83)	**0.003**
	Other	42 (15.00)	20 (7.17)	
Isotype (%)	FLC	41 (14.64)	43 (15.41)	0.820
	IgA	62 (22.14)	71 (25.45)	
	IgG	164 (58.57)	149 (53.41)	
	IgD	1 (0.36)	2 (0.72)	
	Nonsecretory	3 (1.07)	5 (1.79)	
	NA	9 (3.21)	9 (3.23)	
B2M (mean (sd))		4.177 (4.208)	5.290 (6.285)	**0.005**
ALB (mean (sd))		4.093 (0.530)	4.005 (0.628)	0.073
ISS stage (%)	I and II	230 (82.1)	210 (75.3)	**0.047**
	III	50 (17.9)	69 (24.7)	
CRP (mean (sd))		10.327 (18.391)	12.928 (26.779)	0.270
CREAT (mean (sd))		1.239 (1.129)	1.407 (1.399)	0.227
LDH (mean (sd))		170.529 (63.305)	173.430 (68.553)	0.603
HGB (mean (sd))		11.320 (1.825)	11.186 (1.799)	0.382
ASPC (mean (sd))		41.413 (24.065)	43.953 (24.570)	0.229
BMPC (mean (sd))		45.103 (26.527)	47.668 (26.009)	0.256
MRI (mean (sd))		10.314 (13.934)	11.759 (15.102)	0.257
Cytogenetic abnormality	Yes	94 (33.57)	113 (40.50)	0.090
	No	186 (66.43)	166 (59.50)	
Therapy, no (%)	TT2	172 (61.43)	173 (62.01)	0.888
	TT3	108 (38.57)	106 (38.3)	
High CCND1, no (%)		140 (50.18)	140 (50.00)	0.966
High FGFR3, no (%)		142 (50.90)	138 (49.29)	0.703
High LIG4, no (%)		148 (53.05)	132 (47.14)	0.163
High TP53, no (%)		137 (49.10)	143 (51.07)	0.642
High CDK4, no (%)		140 (50.18)	140 (50.00)	0.966
High KRAS, no (%)		131 (46.95)	149 (53.21)	0.139
High NRAS, no (%)		139 (49.82)	141 (50.36)	0.899
High CXCR4, no (%)		158 (56.42)	122 (43.73)	**0.003**
High NOTCH2NL, no (%)		114 (40.71)	166 (59.50)	**<0.001**
High UBE2T, no (%)		117 (41.79)	162 (58.06)	**<0.001**
High IL18R1, no (%)		165 (58.93)	115 (41.22)	**<0.001**
HighMYBL2, no (%)		127 (45.36)	153 (54.84)	**<0.025**

AGE: age at registration (years); B2M: beta-2 microglobulin, mg/l; ALB: albumin, 10 g/l; CRP: c-reactive protein, mg/l; CREAT: creatinine, mg/dl; LDH: lactate dehydrogenase, U/l; HGB: hemoglobin, g/dl; ASPC: aspirate plasma cells (%); BMPC: bone marrow biopsy plasma cells (%); MRI: number of magnetic resonance imaging (MRI)-defined focal lesions (skull, spine, and pelvis); cytogenetic abnormality: an indicator of the detection of cytogenetic abnormalities; TT2: total therapy 2; TT3: total therapy 3; no: number of patients. Bold values mean the differences between two groups are statistically significant.

**Table 2:** Univariate and multivariate cox regression analysis of EFS in GSE24080.

Prognostic parameters	Univariate analysis		Multivariate analysis	
	HR (95% CI)	*p* value	HR (95% CI)	*p* value
SRSF1 (high vs. low)	1.837 (1.347–2.506)	**<0.001**	1.851 (1.353–2.533)	**<0.001**
Age (≥65 vs. <65)	1.047 (0.731–1.500)	0.801	—	—
Gender (female vs. male)	0.951 (0.697–1.297)	0.751	—	—
B2M (≥5.5 vs. <5.5)	1.855 (1.310–2.628)	**0.001**	1.614 (1.132–2.302)	**0.008**
LDH (≥250 vs. <250)	2.633 (1.664–4.169)	**<0.001**	2.590 (1.619–4.143)	**<0.001**
ALB (≥3.5 vs. <3.5)	0.810 (0.525–1.249)	0.340	—	—
MRI (≥2 vs. <2)	1.063 (0.773–1.461)	0.709	—	—

AGE: age at registration (years); B2M: beta-2 microglobulin, mg/l; LDH: lactate dehydrogenase, U/l; ALB: albumin, 10 g/l; MRI: number of magnetic resonance imaging (MRI)-defined focal lesions (skull, spine, and pelvis). HR: hazard ratio and CI: credible interval.

**Table 3:** Univariate and multivariate cox regression analysis of OS in GSE24080.

Prognostic parameters	Univariate analysis		Multivariate analysis	
	HR (95% CI)	*p* value	HR (95% CI)	*p* value
SRSF1 (high vs. low)	1.660 (1.222–2.253)	**0.001**	1.720 (1.254–2.358)	**0.001**
Age (≥65 vs. <65)	1.398 (1.035–1.887)	0.300	—	—
Gender (female vs. male)	1.030 (0.760–1.397)	0.848	—	—
B2M (≥5.5 vs. <5.5)	2.563 (1.868–3.517)	**<0.001**	1.922 (1.351–2.733)	**<0.001**
LDH (≥250 vs. <250)	3.845 (2.624–5.633)	**<0.001**	2.909 (1.919–4.410)	**<0.001**
ALB (≥3.5 vs. <3.5)	0.520 (0.359–0.754)	**0.001**	0.656 (0.444–0.969)	**0.034**
MRI (≥2 vs. <2)	1.660 (1.222–2.253)	**0.001**	1.737 (1.223–2.467)	**0.002**

AGE: age at registration (years); B2M: beta-2 microglobulin, mg/l; LDH: lactate dehydrogenase, U/l; ALB: albumin, 10 g/l; MRI: number of magnetic resonance imaging (MRI)-defined focal lesions (skull, spine, and pelvis). HR: hazard ratio and CI: credible interval. Bold values mean the differences between two groups (like SRSF1-high vs. SRSF1-low) are statistically significant.

## Data Availability

Publicly available datasets were used in this study. These data can be found here: Gene Expression Omnibus (GEO) repository (https://www.ncbi.nlm.nih.gov/geo/). The data that support the findings of this study are available from the corresponding author upon reasonable request.

## Supplementary Materials

Supplementary Materials Supplemental Table 1. Differential expressed genes between the SRSF1high and the SRSF1low groups. Supplementary Table 2. Top 20 terms of GO analysis of differential expressed genes. Supplementary Table 3. Top 20 terms of KEGG pathway analysis of differential expressed genes. Supplementary Figure 1. SRSF1 expression levels at different 1q21 amplification in 248 MM patients of GSE2658. Supplementary Figure 2. The Kaplan–Meier curve shows the prognostic value of the SRSF1 expression levels for MM patients categorized by B2M ≤ 3.5 (mg/l) (A, D), 3.5 < B2M < 5.5 (mg/l) (B, E), and B2M ≥ 5.5 (mg/l) (C, F). Supplementary Figure 3. The Kaplan–Meier curve shows the prognostic value of the SRSF1 expression levels for MM patients categorized by LDH < 250 (U/l) (A, C) and LDH ≥ 250 (U/l) (B, D). Supplementary Figure 4. GSEA showed that hematopoietic cell lineage (A), cell receptor signaling pathway (B), TNFa signaling via NFKB (C), inflammatory response (D), complement (E), and coagulation (F) were significantly enriched in the SRSF1low group.
