# Supplementary material for: Increased Expression of SRSF1 Predicts Poor Prognosis in Multiple Myeloma
Source: J Oncol. 2023 May 10;2023:9998927. doi: 10.1155/2023/9998927 (PMC10191755; doi:10.1155/2023/9998927)
Supplement: Supplementary Materials — Supplemental Table 1. Differential expressed genes between the SRSF1high and the SRSF1low groups. Supplementary Table 2. Top 20 terms of GO analysis of differential expressed genes. Supplementary Table 3. Top 20 terms of KEGG pathway analysis of differential expressed genes. Supplementary Figure 1. SRSF1 expression levels at different 1q21 amplification in 248 MM patients of GSE2658. Supplementary Figure 2. The Kaplan–Meier curve shows the prognostic value of the SRSF1 expression levels for MM patients categorized by B2M ≤ 3.5 (mg/l) (A, D), 3.5 < B2M < 5.5 (mg/l) (B, E), and B2M ≥ 5.5 (mg/l) (C, F). Supplementary Figure 3. The Kaplan–Meier curve shows the prognostic value of the SRSF1 expression levels for MM patients categorized by LDH < 250 (U/l) (A, C) and LDH ≥ 250 (U/l) (B, D). Supplementary Figure 4. GSEA showed that hematopoietic cell lineage (A), cell receptor signaling pathway (B), TNFa signaling via NFKB (C), inflammatory response (D), complement (E), and coagulation (F) were significantly enriched in the SRSF1low group. [file 9998927.f1.pdf]

Supplemental Table 1. Differential expressed genes between the SRSF1^{high} and the SRSF1^{low} groups.

Gene	log_2FC	t	P-Value	adj.P-Value
MIR142	0.74055	6.55985	1.22E-10	6.47E-08
AC004941.5	0.71396	5.83016	9.35E-09	2.07E-06
INHBE	0.69739	3.88298	0.000115	0.003023
LINC01021	0.67628	4.57487	5.87E-06	0.000333
LOC100129917	0.66612	5.16442	3.35E-07	3.68E-05
EIF3C	0.66368	6.92083	1.23E-11	1.11E-08
SLC20A1	0.65201	6.65325	6.82E-11	4.10E-08
EMC3-AS1	0.64659	5.55547	4.28E-08	7.08E-06
LOC100134822	0.64512	5.42139	8.80E-08	1.25E-05
BC045784	0.62852	4.7656	2.40E-06	0.000167
LINC00328	0.6192	4.91313	1.18E-06	9.81E-05
HJURP	0.61576	5.21213	2.63E-07	3.14E-05
CTB-181H17.1	0.61253	6.11972	1.76E-09	5.52E-07
KIF21B	0.6001	4.01938	6.63E-05	0.002054
SKA1	0.59651	4.7375	2.74E-06	0.000182
RP11-480A16.1	0.57599	5.20026	2.79E-07	3.23E-05
RP1-102H19.8	0.5723	4.92855	1.09E-06	9.38E-05
RP11-124L9.5	0.5652	4.80539	1.99E-06	0.000147
TACC3	0.5644	4.87487	1.42E-06	0.000113
ZNF559	0.55644	3.62061	0.000321	0.00628
LOC100132352	0.55318	5.06205	5.63E-07	5.62E-05
HPDL	0.55125	4.56823	6.05E-06	0.000337
CRIM1	0.54821	2.97252	0.00308	0.031927
FOXM1	0.54518	4.02271	6.54E-05	0.002038
ZNF569	0.54448	4.43914	1.09E-05	0.000526
AZGP1	0.5317	2.80017	0.005284	0.046137
SCN3A	0.53159	2.29829	0.021912	0.120784
ZFP30	0.52842	3.32282	0.000949	0.013871
SNORA21	0.52295	5.67914	2.17E-08	3.98E-06
C1orf112	0.52197	4.07003	5.38E-05	0.001766
PHF19	0.52042	5.46346	7.03E-08	1.04E-05
LOC100134445	0.51943	6.99161	7.75E-12	8.39E-09
RPL36A	0.51435	6.08822	2.12E-09	6.55E-07
AGAP4	0.51404	7.49845	2.54E-13	6.77E-10
ASPM	0.51127	5.75884	1.40E-08	2.82E-06
PXMP2	0.5109	5.20781	2.69E-07	3.16E-05
TOP2A	0.50888	5.13185	3.96E-07	4.20E-05
ANKLE1	0.50671	4.41759	1.20E-05	0.000564
CENPM	0.5035	3.95649	8.58E-05	0.002454
RRN3P2	0.50224	4.31802	1.86E-05	0.000792
RRM2	0.50115	4.58102	5.71E-06	0.000326
LOC100271840	0.49762	3.9203	9.94E-05	0.002737
NUF2	0.49539	4.16191	3.65E-05	0.001322

HIST1H2AM	0.49446	3.77678	0.000176	0.004116
AF090939	0.49191	5.24165	2.26E-07	2.73E-05
BC032415	0.4909	5.44446	7.78E-08	1.14E-05
POLE2	0.48906	4.48479	8.86E-06	0.000449
BC022892	0.48599	4.57397	5.89E-06	0.000333
NOMO3	0.48546	5.19243	2.91E-07	3.30E-05
NVL	0.48294	3.71246	0.000226	0.004957
MGC12488	0.48066	3.71698	0.000222	0.004896
KIF14	0.47538	5.69028	2.04E-08	3.82E-06
C1orf106	0.47316	3.48509	0.00053	0.009198
PMS2P5	0.47162	7.02739	6.12E-12	6.98E-09
NCAPH	0.47161	3.91704	0.000101	0.002751
DTL	0.46913	5.31762	1.52E-07	1.95E-05
LOC101060510	0.46619	3.81719	0.00015	0.003653
NOTCH2NL	0.46593	3.96583	8.26E-05	0.0024
LOC285812	0.46504	5.27903	1.86E-07	2.28E-05
HNRNPU-AS1	0.4624	7.03058	6.00E-12	6.98E-09
AP000347.2	0.4573	4.73057	2.84E-06	0.000186
RP11-50B3.4	0.45658	6.35483	4.32E-10	1.70E-07
LINC01410	0.4558	4.85368	1.57E-06	0.000124
CENPK	0.45544	4.41397	1.22E-05	0.000569
STK11IP	0.4513	4.335	1.73E-05	0.000744
SHCBP1	0.45058	4.07536	5.26E-05	0.001743
ANKRD36B	0.45029	5.65127	2.54E-08	4.46E-06
ANKRD36C	0.44966	5.47547	6.59E-08	1.00E-05
AF131215.4	0.44867	3.75125	0.000194	0.004441
LOC100505874	0.44667	3.73932	0.000203	0.004579
GINS1	0.44436	4.75505	2.53E-06	0.000173
STC2	0.44394	4.96927	8.94E-07	8.17E-05
RP3-428L16.2	0.44273	3.44502	0.000614	0.010198
KIF4A	0.44229	4.02934	6.36E-05	0.001993
BC030152	0.44164	4.90168	1.25E-06	0.000102
ARF1	0.44065	4.17471	3.46E-05	0.001273
CTAG2	0.43857	3.48997	0.000521	0.009081
LOC101928700	0.43835	4.09683	4.81E-05	0.001636
LOC399491	0.43823	4.7415	2.69E-06	0.000181
RP11-803D5.4	0.43738	4.11082	4.53E-05	0.00156
LOC81691	0.43607	5.38268	1.08E-07	1.47E-05
KIF20A	0.43362	3.86417	0.000124	0.003213
RP11-271C24.3	0.43359	7.59838	1.26E-13	3.91E-10
AK025288	0.43302	4.63303	4.48E-06	0.000271
MYBL2	0.43271	3.2039	0.001433	0.018756
CLTC-IT1	0.42904	4.38827	1.37E-05	0.00062
LINC00403	0.42648	2.68748	0.007413	0.058452
LOC101927709	0.42513	2.98738	0.002937	0.030882
NLRC3	0.42373	2.96075	0.003199	0.032859
LOC101929004	0.42367	3.84815	0.000133	0.003376

RP5-1074L1.4	0.42367	6.15173	1.46E-09	4.71E-07
C3orf67	0.42361	3.0824	0.002154	0.024797
LOC100190986	0.4207	6.26391	7.48E-10	2.70E-07
LOC101928433	0.41937	2.81702	0.005018	0.044545
LOC284513	0.41641	6.02672	3.03E-09	8.75E-07
LRRC69	0.41549	4.56722	6.08E-06	0.000337
LOC145474	0.41518	3.43053	0.000647	0.01056
LOC100272216	0.41517	6.69007	5.41E-11	3.35E-08
RP11-138A9.1	0.41346	5.94256	4.93E-09	1.21E-06
BD495725	0.41196	3.92808	9.63E-05	0.00268
RP3-508I15.21	0.4118	3.79915	0.000161	0.003863
MIR34A	0.41049	3.14363	0.001757	0.021484
LOC102723721	0.41042	4.34998	1.62E-05	0.000712
FTX	0.41026	4.38485	1.39E-05	0.000625
STAG3L3	0.40779	5.13886	3.82E-07	4.10E-05
MSH2	0.40767	5.42739	8.52E-08	1.23E-05
DSCR8	0.40742	2.78614	0.005514	0.047626
SMAD1	0.40697	2.1019	0.036007	0.167732
ANKS3	0.4069	3.65711	0.000279	0.005715
LOC101929964	0.40653	4.09331	4.88E-05	0.001647
CCAR1	0.40531	7.71115	5.71E-14	2.06E-10
E2F8	0.40378	4.64554	4.23E-06	0.000257
SND1-IT1	0.40324	5.52618	5.02E-08	8.05E-06
ARHGEF1	0.40322	3.96778	8.20E-05	0.002385
LOC101927330	0.40192	3.5544	0.000411	0.007537
PFAS	0.40148	6.46513	2.20E-10	1.01E-07
ZNF692	0.4011	8.39844	3.71E-16	2.68E-12
HK2	0.40088	2.31511	0.020967	0.11728
AP001462.6	0.40059	3.46185	0.000577	0.009766
ASZ1	0.40037	3.19025	0.001501	0.019283
ZNF76	0.39967	3.9314	9.50E-05	0.002654
MAGEB18	0.39925	2.53127	0.011637	0.078887
NEK2	0.39879	4.29707	2.04E-05	0.000853
PLK4	0.39839	4.23606	2.66E-05	0.001041
KCNK12	0.39823	2.82275	0.00493	0.043991
ZNF883	0.39805	3.62358	0.000317	0.006217
PMS2L2	0.39725	3.97289	8.03E-05	0.002355
AGAP9	0.3972	4.14579	3.91E-05	0.001392
AX747652	0.39688	3.55886	0.000404	0.007451
PBK	0.39663	4.3019	2.00E-05	0.000841
HTR2C	0.39626	3.83169	0.000142	0.003502
MED6	0.39465	6.01636	3.22E-09	9.05E-07
SEC14L1P1	0.39337	3.60852	0.000335	0.006497
UHRF1	0.39308	3.56242	0.000399	0.007378
NUDT9P1	0.39258	3.9952	7.33E-05	0.002206
ANKZF1	0.39087	3.94122	9.13E-05	0.002581

LL22NC03-N14H11.1	0.39082	3.3983	0.000726	0.011481
BRIP1	0.38982	4.18723	3.28E-05	0.00122
HIST1H4J	0.38845	3.09707	0.002052	0.023989
STAM-AS1	0.38844	3.45385	0.000594	0.009984
C10orf40	0.38805	3.02421	0.002607	0.028561
BUB1B	0.3878	4.30258	1.99E-05	0.000841
MEST	0.38774	4.02649	6.44E-05	0.002012
DEPDC1B	0.3876	3.41309	0.000689	0.010996
RPS16P5	0.38682	3.43761	0.00063	0.010387
SSX1	0.38611	3.15723	0.001678	0.020753
TCERG1	0.38583	6.97119	8.86E-12	9.13E-09
PKD1P1	0.38567	3.6604	0.000276	0.005678
BC042590	0.38554	3.43909	0.000627	0.010352
TENM2	0.38445	3.39207	0.000743	0.011659
MINOS1P1	0.38425	4.28948	2.11E-05	0.000871
D2HGDH	0.38211	3.99538	7.32E-05	0.002206
FLJ41455	0.38199	3.09084	0.002095	0.024321
GTF2H2B	0.38144	6.04721	2.69E-09	7.98E-07
TOX2	0.38143	2.2953	0.022084	0.121268
BTBD18	0.38056	3.82717	0.000144	0.003541
LOC101927196	0.37979	3.1046	0.002001	0.023616
OIP5	0.37934	3.97103	8.09E-05	0.002366
CKAP2L	0.37932	3.65747	0.000279	0.005715
UBE2T	0.37834	4.22167	2.83E-05	0.001088
HELLS	0.37828	5.80532	1.08E-08	2.26E-06
SPC25	0.37807	4.20047	3.10E-05	0.001162
GSTP1	-0.37881	-4.31644	1.87E-05	0.000796
C10orf10	-0.37929	-2.71364	0.006859	0.055438
APOC1	-0.38108	-3.02408	0.002608	0.028561
CTC-360P9.3	-0.38133	-4.10216	4.70E-05	0.001605
CSF2RB	-0.38217	-3.04603	0.002428	0.027222
CYP26A1	-0.38227	-3.29577	0.001043	0.014882
LOC101929272	-0.38231	-3.62774	0.000312	0.006135
ST6GALNAC6	-0.38417	-3.31717	0.000968	0.014088
TNFSF8	-0.38492	-2.74531	0.006239	0.051898
RGS2	-0.38503	-3.18648	0.00152	0.019445
NCF4	-0.38933	-4.96116	9.30E-07	8.36E-05
LOC101928612	-0.39003	-3.8364	0.000139	0.003476
MAFB	-0.39134	-2.72661	0.006599	0.053855
ACP5	-0.39198	-3.72329	0.000216	0.004824
MARCKS	-0.39241	-3.46222	0.000577	0.009764
TNFSF10	-0.39277	-3.16651	0.001627	0.020381
NFKBIE	-0.3949	-4.3929	1.34E-05	0.000612
RNASE3	-0.39611	-2.74541	0.006237	0.051898
CTSH	-0.3966	-2.57015	0.010422	0.073818
ABCG2	-0.39737	-2.82498	0.004897	0.043864

SPNS3	-0.39748	-2.50982	0.012359	0.082063
UBE2QL1	-0.39831	-2.87436	0.004202	0.039282
RALYL	-0.40002	-2.39965	0.016736	0.100071
LOC100996425	-0.4001	-4.23795	2.64E-05	0.001034
LILRB5	-0.40316	-2.99682	0.002849	0.030295
FKBP1B	-0.40479	-3.50457	0.000494	0.008722
ZC3H12D	-0.40594	-2.87753	0.004161	0.039015
LOC101929143	-0.40688	-3.50752	0.000489	0.008656
MANSC1	-0.40717	-3.49664	0.000508	0.008906
CCR5	-0.40724	-2.41494	0.016056	0.097109
HOXC9	-0.40784	-3.72327	0.000217	0.004824
LOC100131662	-0.40916	-3.02879	0.002568	0.028285
CEACAM8	-0.40918	-2.76969	0.005797	0.049219
HMOX1	-0.41236	-2.95797	0.003227	0.033006
ELANE	-0.41244	-3.14164	0.001769	0.021611
FGF2	-0.41385	-3.67716	0.000259	0.005403
STAP1	-0.41388	-3.55654	0.000408	0.007497
SELM	-0.41593	-4.78862	2.15E-06	0.000154
EPX	-0.4161	-3.35863	0.000836	0.012694
PLBD1	-0.41782	-3.47443	0.000551	0.009455
IGHM	-0.41997	-3.06435	0.002286	0.025999
BC034416	-0.42158	-3.97991	7.80E-05	0.002301
GADD45A	-0.42177	-4.05066	5.83E-05	0.001874
TRPM4	-0.42324	-2.18126	0.029578	0.147666
IL18	-0.42679	-3.054	0.002365	0.026628
TMEM56	-0.43008	-3.99951	7.20E-05	0.002186
NOL4	-0.4301	-2.56508	0.010574	0.074479
P2RY6	-0.43116	-2.91405	0.00371	0.036298
SYNE4	-0.43133	-4.37416	1.45E-05	0.000652
CD74	-0.43656	-6.25883	7.71E-10	2.74E-07
SLC46A3	-0.43712	-3.39743	0.000728	0.011487
DCLK1	-0.43979	-3.10128	0.002024	0.023799
CXCL12	-0.44077	-3.78412	0.000171	0.004029
LAMP3	-0.44171	-4.49619	8.41E-06	0.000433
CD14	-0.44292	-2.9662	0.003143	0.032394
GLCE	-0.44334	-4.56923	6.02E-06	0.000337
PPAP2C	-0.44344	-2.13713	0.033019	0.158471
UNC119	-0.44425	-4.67446	3.69E-06	0.000232
TMEM45A	-0.4472	-4.12678	4.24E-05	0.001484
LOC102724362	-0.45085	-3.93395	9.40E-05	0.002636
RASGRP1	-0.454	-2.11701	0.034698	0.163858
PLA2G2D	-0.45606	-4.42679	1.15E-05	0.000548
NR3C2	-0.45668	-3.65703	0.000279	0.005715
MPEG1	-0.45832	-4.01963	6.62E-05	0.002054
PRR15	-0.45923	-2.31051	0.021222	0.118099
TUBB2A	-0.4608	-3.27971	0.001104	0.015475
CPQ	-0.46137	-5.46622	6.93E-08	1.03E-05

PPBP	-0.46141	-2.43005	0.015409	0.094838
AMIGO2	-0.46292	-3.84546	0.000134	0.0034
SCGB1A1	-0.46318	-4.21209	2.95E-05	0.001121
LINC00324	-0.46456	-4.93186	1.07E-06	9.27E-05
DPEP1	-0.46514	-2.96105	0.003196	0.032854
LOC100996286	-0.46537	-4.56974	6.01E-06	0.000337
TMCO2	-0.46589	-4.24909	2.51E-05	0.000992
LAG3	-0.46687	-2.87224	0.00423	0.039425
CD163	-0.46806	-3.57634	0.000379	0.007096
CCRL2	-0.46905	-3.28625	0.001079	0.015212
NCF2	-0.46936	-3.07592	0.002201	0.025225
SOX14	-0.46967	-4.15584	3.75E-05	0.001354
LOC100507562	-0.47165	-4.05742	5.67E-05	0.001831
CCL18	-0.47636	-3.5788	0.000375	0.007046
TIMD4	-0.47841	-3.60055	0.000346	0.006665
NOD2	-0.47855	-3.96403	8.32E-05	0.002409
APOL3	-0.48128	-3.63606	0.000302	0.006036
SIT1	-0.4834	-3.19078	0.001498	0.019263
NXPE4	-0.48907	-4.41719	1.20E-05	0.000564
GNG11	-0.49098	-3.93942	9.20E-05	0.002595
C1QC	-0.49297	-3.08768	0.002117	0.024483
PLEKHF1	-0.49379	-4.21889	2.86E-05	0.001093
IGSF11	-0.4948	-3.73233	0.000209	0.00469
IL18R1	-0.49488	-4.77634	2.28E-06	0.000161
EYA2	-0.49585	-2.73222	0.006489	0.0532
SYNGR3	-0.49633	-3.12896	0.001845	0.022264
ADTRP	-0.50134	-4.56487	6.15E-06	0.000339
GPHA2	-0.50598	-4.26091	2.39E-05	0.000956
C11orf96	-0.50907	-2.68881	0.007384	0.058306
CTGF	-0.52266	-2.32111	0.020638	0.116007
IGK	-0.52609	-4.01027	6.89E-05	0.002115
ATXN8OS	-0.5283	-4.09184	4.91E-05	0.001655
PLAT	-0.53034	-4.46601	9.64E-06	0.000479
SERPINI1	-0.53343	-4.46576	9.65E-06	0.000479
ICAM4	-0.53684	-3.64503	0.000292	0.005902
PTPRZ1	-0.54619	-2.89604	0.003926	0.037549
PRG2	-0.54916	-3.13965	0.00178	0.021722
MLIP	-0.55058	-3.68246	0.000253	0.005367
ISL2	-0.56446	-2.4467	0.014723	0.091762
CD5L	-0.56763	-4.83434	1.73E-06	0.000133
HLA-DRA	-0.57133	-3.1602	0.001662	0.020641
ELOVL7	-0.57142	-3.48718	0.000526	0.009139
LOC100293211	-0.5725	-3.74848	0.000196	0.004466
VCAM1	-0.58662	-3.85973	0.000127	0.003254
C1QB	-0.59581	-3.70087	0.000236	0.005126
IGHV3-54	-0.60398	-3.96556	8.27E-05	0.0024
MAST1	-0.60547	-4.87717	1.40E-06	0.000113

CPVL	-0.61265	-3.8819	0.000116	0.003033
QPCT	-0.62142	-3.67729	0.000258	0.005403
SULF2	-0.62303	-3.18001	0.001554	0.019711
PLEKHO1	-0.654	-3.82831	0.000144	0.00354
BIRC3	-0.65768	-4.89198	1.31E-06	0.000106
C1QA	-0.6995	-3.93926	9.20E-05	0.002595
Igk	-0.73808	-5.35038	1.28E-07	1.68E-05
GBA3	-0.74999	-4.64818	4.18E-06	0.000256
CTSW	-0.84207	-3.97868	7.84E-05	0.002309
KIT	-0.991	-4.46451	9.70E-06	0.000481

Supplementary Table 2. Top 20 terms of GO analysis of differential expressed genes.

GO	Description	logP-Value	Z-score	Gene Number	Gene Count	Gene Ratio (%)
GO:0051301	cell division	-8.9	8.3	213	22	10
GO:0006954	inflammatory response	-6.9	6.8	213	22	10
GO:0050778	positive regulation of immune response	-4.1	4.8	213	17	8
GO:0007059	chromosome segregation	-8.1	8.5	213	16	7.5
GO:0002274	myeloid leukocyte activation	-4.3	5	213	16	7.5
GO:0002366	leukocyte activation involved in immune response	-3.9	4.6	213	16	7.5
GO:0002263	cell activation involved in immune response	-3.9	4.6	213	16	7.5
GO:0046649	lymphocyte activation	-3.7	4.4	213	16	7.5
GO:0001817	regulation of cytokine production	-3.5	4.2	213	16	7.5
GO:0002697	regulation of immune effector process	-5.5	6.2	213	15	7
GO:0002250	adaptive immune response	-3.4	4.2	213	15	7
GO:0009617	response to bacterium	-3.3	4.1	213	15	7
GO:0051345	positive regulation of hydrolase activity	-3	3.8	213	15	7
GO:0045055	regulated exocytosis	-3	3.8	213	15	7
GO:0045859	regulation of protein kinase activity	-2.9	3.7	213	15	7
GO:0000280	nuclear division	-5.1	5.9	213	14	6.6
GO:0048285	organelle fission	-4.6	5.4	213	14	6.6
GO:0050900	leukocyte migration	-4.4	5.2	213	14	6.6
GO:0002694	regulation of leukocyte activation	-3.7	4.6	213	14	6.6
GO:0000226	microtubule cytoskeleton organization	-3.6	4.4	213	14	6.6

Supplementary Table 3. Top 20 terms of KEGG pathway analysis of differential expressed genes.

KEGG pathway	Description	LogP-Value	Z-score	Gene Number	Gene Count	Gene Ratio (%)
hsa04060	Cytokine-cytokine receptor interaction	-3	4.2	213	8	3.8
hsa05322	Systemic lupus erythematosus	-4.1	6	213	7	3.3
hsa05202	Transcriptional misregulation in cancer	-2.3	3.6	213	6	2.8
hsa04610	Complement and coagulation cascades	-3.5	5.7	213	5	2.3
hsa04210	Apoptosis	-2.4	3.9	213	5	2.3
hsa05418	Fluid shear stress and atherosclerosis	-2.3	3.7	213	5	2.3
hsa04145	Phagosome	-2	3.3	213	5	2.3
hsa05321	Inflammatory bowel disease (IBD)	-2.8	4.9	213	4	1.9
hsa01524	Platinum drug resistance	-2.6	4.7	213	4	1.9
hsa05150	Staphylococcus aureus infection	-2.5	4.4	213	4	1.9
hsa05133	Pertussis	-2.5	4.4	213	4	1.9
hsa05323	Rheumatoid arthritis	-2.2	3.8	213	4	1.9
hsa04064	NF-kappa B signaling pathway	-2	3.6	213	4	1.9
ko04668	TNF signaling pathway	-2	3.5	213	4	1.9
hsa05310	Asthma	-2.8	5.7	213	3	1.4
hsa05020	Prion diseases	-2.6	5.3	213	3	1.4
ko05134	Legionellosis	-2.1	4	213	3	1.4

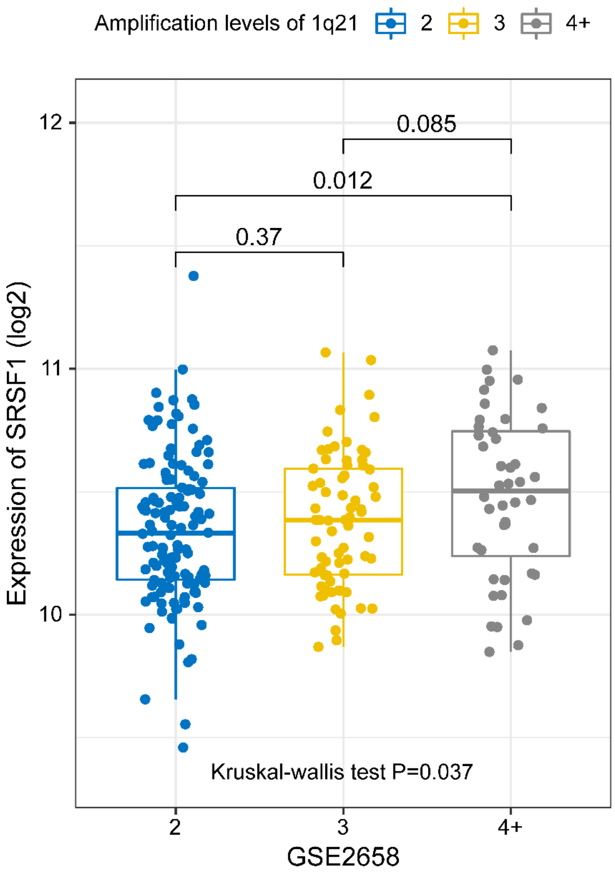

Supplementary Figure 1. SRSF1 expression levels at different 1q21 amplification in 248 MM patients of GSE2658. p = 0.037, Kruskal-wallis test.

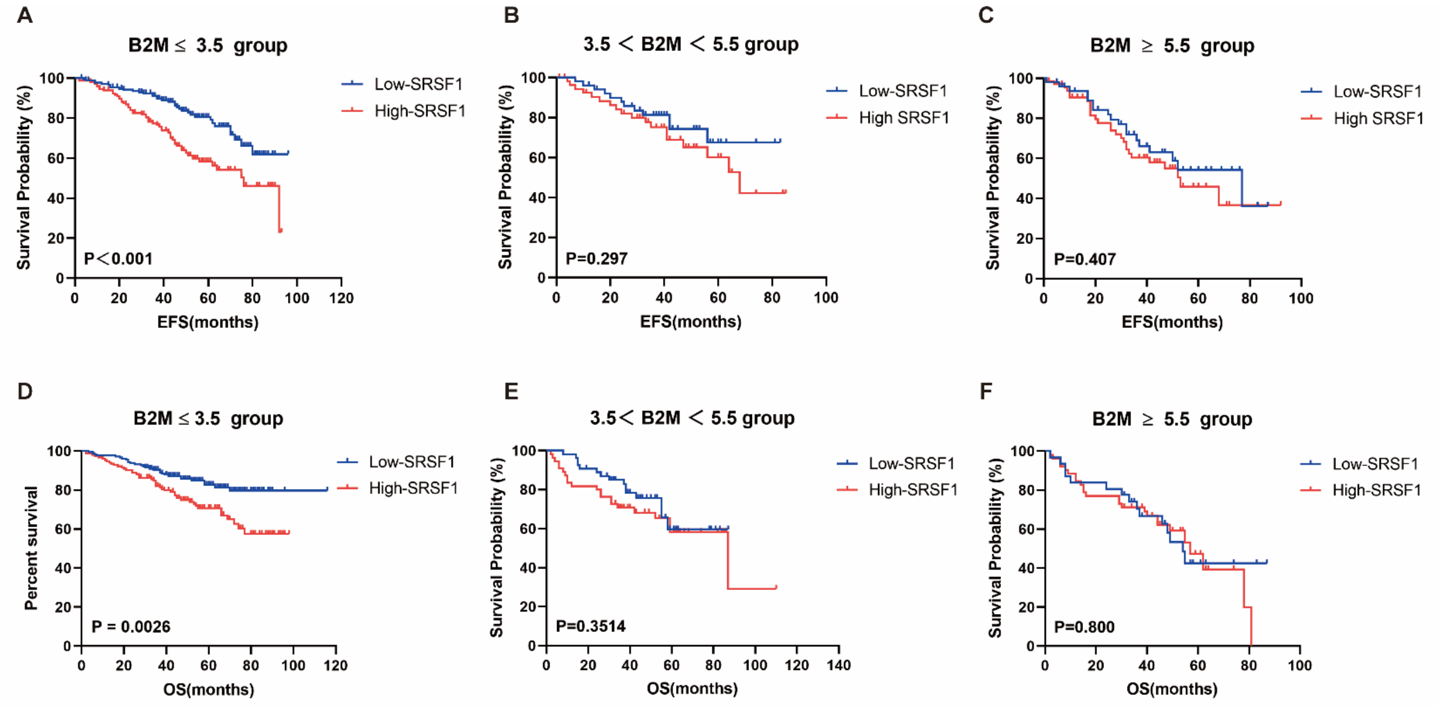

Supplementary Figure 2. The Kaplan-Meier curve shows the prognostic value of the SRSF1 expression levels for MM patients categorized by B2M⩽3.5(mg/l) (**A, D**), 3.5 < B2M < 5.5(mg/l) (**B, E**), and B2M⩾5.5(mg/l) (**C, F**). Abbreviation: B2M: beta-2 microglobulin; EFS: event free survival; OS: overall survival.

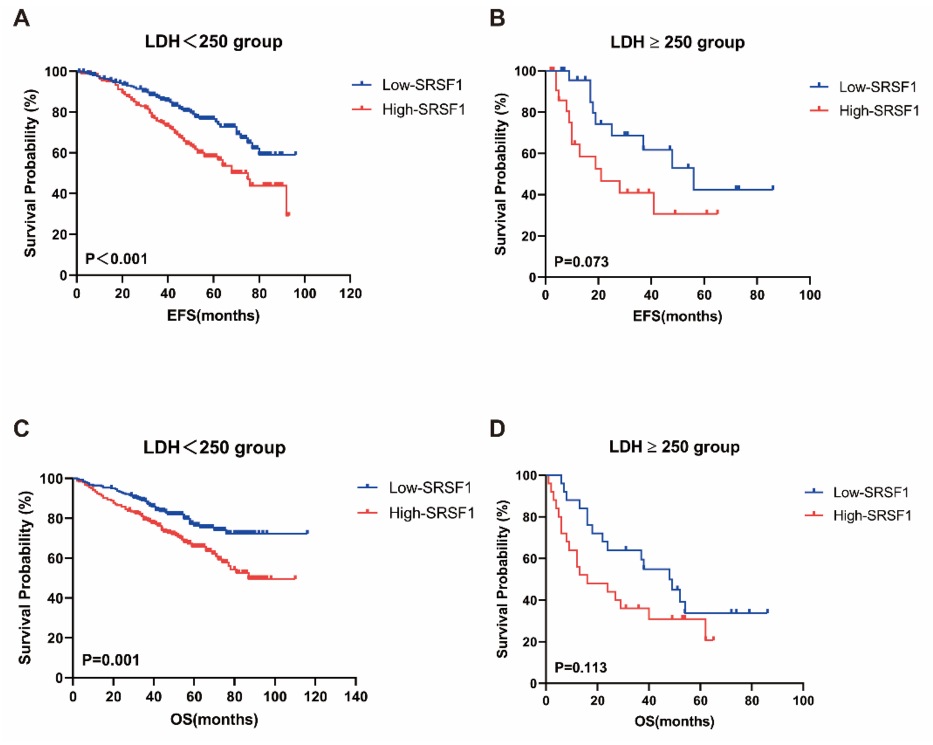

Supplementary Figure 3. The Kaplan-Meier curve shows the prognostic value of the SRSF1 expression levels for MM patients categorized by LDH < 250(U/l) (**A, C**) and LDH⩾250(U/l) (**B, D**). Abbreviation: LDH: lactate dehydrogenase; EFS: event free survival; OS: overall survival.

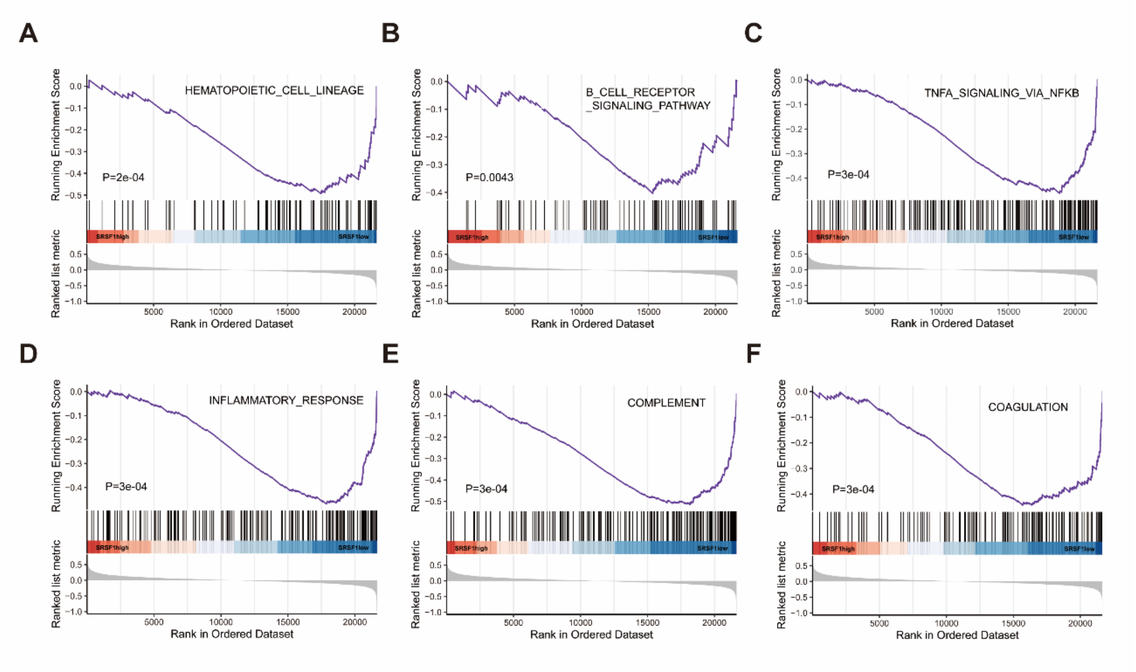

Supplementary Figure 4. GSEA showed that hematopoietic cell lineage(**A**), B cell receptor signaling pathway(**B**), TNFa signaling via NFKB(**C**), inflammatory response(**D**), complement(**E**), and coagulation(**F**) were significantly enriched in the $SRSF1^{low}$ group.
